# Discrimination of Lipogenic or Glucogenic Diet Effects in Early-Lactation Dairy Cows Using Plasma Metabolite Abundances and Ratios in Combination with Machine Learning

**DOI:** 10.3390/metabo14040230

**Published:** 2024-04-17

**Authors:** Xiaodan Wang, Sanjeevan Jahagirdar, Wouter Bakker, Carolien Lute, Bas Kemp, Ariette van Knegsel, Edoardo Saccenti

**Affiliations:** 1 Adaptation Physiology Group, Department of Animal Sciences, Wageningen University & Research, 6700 AH Wageningen, The Netherlands; wangxd_dr@163.com (X.W.); bas.kemp@wur.nl (B.K.); ariette.vanknegsel@wur.nl (A.v.K.); 2 Laboratory of Systems and Synthetic Biology, Wageningen University & Research, 6700 EJ Wageningen, The Netherlands; jahagirdar.sanjeevan@gmail.com; 3 Institute of Food Science and Technology, Chinese Academy of Agricultural Sciences, Beijing 100193, China; 4 Division of Toxicology, Wageningen University & Research, 6700 EA Wageningen, The Netherlands; wouter.bakker@wur.nl

**Keywords:** energy unbalance, machine learning, plasma, milk, systems biology

## Abstract

During early lactation, dairy cows have a negative energy balance since their energy demands exceed their energy intake: in this study, we aimed to investigate the association between diet and plasma metabolomics profiles and how these relate to energy unbalance of course in the early-lactation stage. Holstein-Friesian cows were randomly assigned to a glucogenic (*n* = 15) or lipogenic (*n* = 15) diet in early lactation. Blood was collected in week 2 and week 4 after calving. Plasma metabolite profiles were detected using liquid chromatography–mass spectrometry (LC-MS), and a total of 39 metabolites were identified. Two plasma metabolomic profiles were available every week for each cow. Metabolite abundance and metabolite ratios were used for the analysis using the XGboost algorithm to discriminate between diet treatment and lactation week. Using metabolite ratios resulted in better discrimination performance compared with the metabolite abundances in assigning cows to a lipogenic diet or a glucogenic diet. The quality of the discrimination of performance of lipogenic diet and glucogenic diet effects improved from 0.606 to 0.753 and from 0.696 to 0.842 in week 2 and week 4 (as measured by area under the curve, AUC), when the metabolite abundance ratios were used instead of abundances. The top discriminating ratios for diet were the ratio of arginine to tyrosine and the ratio of aspartic acid to valine in week 2 and week 4, respectively. For cows fed the lipogenic diet, choline and the ratio of creatinine to tryptophan were top features to discriminate cows in week 2 vs. week 4. For cows fed the glucogenic diet, methionine and the ratio of 4-hydroxyproline to choline were top features to discriminate dietary effects in week 2 or week 4. This study shows the added value of using metabolite abundance ratios to discriminate between lipogenic and glucogenic diet and lactation weeks in early-lactation cows when using metabolomics data. The application of this research will help to accurately regulate the nutrition of lactating dairy cows and promote sustainable agricultural development.

## 1. Introduction

Early lactation is one of the most challenging periods dairy cows face. During this period, cows need to cope with the nutritional demands of lactation, while also coping with the demands of reproductive system recovery and body maintenance [1]. During early lactation, dairy cows have a negative energy balance since their energy demands exceed their energy intake [2]. At this time, cows mobilize body fat, protein, and minerals to meet the requirements for milk production [3]. The energy balance is not only related to metabolic disturbances and diseases in dairy cows [4,5], but also to milk yield and composition [6,7,8].

Regulating dairy cow lactation through diet is an important approach in lactation management. The effects of nutrient restriction in dairy cow diets can be reflected in the decreased concentrations of glutamate and uric acid in milk [9]. In a previous study, we found that a glucogenic diet improved the energy balance in dairy cows compared with a lipogenic diet, associated with lower plasma non-esterified fatty acids (NEFAs) and beta-hydroxybutyrate (BHB) concentrations and liver triglyceride concentrations and higher plasma insulin concentrations [10].

Besides the conventional determined plasma variables described above, metabolomics approaches have also been widely used in the field of dairy science. Metabolomics approaches identify and quantify small-molecule metabolites from biological samples like plasma or urine, by mass spectrometry (MS) or nuclear magnetic resonance [11,12,13]. After the detection of these small-molecule metabolites, mining effective information from these metabolomics data to screen potential biomarkers is the key to metabolomics data analyses. Machine learning is a field devoted to building predictive models from multidimensional datasets, and has become a data analysis technique for mining effective information from complex datasets [14]. Integrating metabolomics approaches and machine learning algorithms can extract effective information from complex and massive metabolomics datasets, which is an important approach in systems biology research [15]. Based on the prediction model established by a machine learning algorithm, the glucose, total protein, and globulin in milk were shown to predict the corresponding metabolites in cow blood [16].

Moreover, besides absolute and relative abundances of metabolites, ratios between metabolites have also been applied in biomedical fields as specific biomarkers [17]. The ratios between molecules, not only their concentration, are relevant [18] as molecules behave in a coordinated way through metabolic pathways. Changes in their association patterns can provide information on the reregulation of biochemical reaction networks and metabolic pathways. Such interactions between metabolites can provide information at the level of biological systems rather than a single metabolic pathway [19]. In dairy science, the glycerophosphocholine to phosphorylcholine has been suggested as a potential marker to identify whether cows are at potential risk of ketosis during the first 4 weeks of lactation [18,20].

In this study, we combine plasma metabolomic data and machine learning algorithms to investigate how different dietary regimens affect the energy metabolism in dairy cows in early lactation. Two dietary treatments were considered (lipogenic and glucogenic diet) and cows were sampled on different occasions for blood, which was subsequently metabolomic-analyzed via LC-MS. The overarching goal was to understand the association between diet and metabolomics profiles and how these relate to an energy unbalance of course in the early-lactation stage. This research aligns well with the fulfillment of the Sustainable Development Goals (SDGs) [21], and the results will provide new ideas for promoting sustainable agriculture and sustainable production models.

## 2. Materials and Methods

### 2.1. Animals and Experimental Design

**Figure 1:**
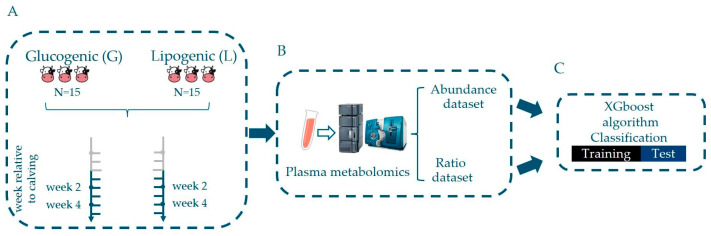
An overview of the experimental setup and data processing. (**A**) Plasma samples were obtained at lactation week 2 and week 4 relative to calving from cows fed a glucogenic or a lipogenic diet. (**B**) The metabolomics analysis of plasma was performed using LC-MS. Metabolite abundance data were used to calculate metabolite ratios. (**C**) The metabolite abundances and the metabolite ratios were used to discriminate a dietary effect (comparing lipogenic and glucogenic diet at lactation week 2 and week 4) and diet-specific effect by comparing plasma profiles at lactation week 2 with those at week 4. Discriminant models were built using the XGboost algorithm.

The Institution Animal Care and Use Committee of Wageningen University (Wageningen, The Netherlands) approved the experimental protocol (registration number 2010026). The experimental design and diets were described earlier [10]. In short, Holstein-Friesian cows (n = 30, 2nd, 3rd, or ≥4th parity) were selected from the Dairy Campus research herd and randomly assigned to early-lactation diets (n = 15 and n = 15 for lipogenic and glucogenic, respectively) Figure 1.

### 2.2. Characteristics of Lipogenic and Glucogenic Diets

Diet composition was described earlier [10]. In short, from week 3 prepartum onwards, all cows were fed 1 kg/day of the experimental concentrate (lipogenic or glucogenic). Post-calving, the supply of experimental concentrates was 1 kg and was incrementally increased by 0.5 kg/day until the experimental concentrate supply reached 8.5 kg/day at d 17 post-calving. Experimental concentrates were provided by a computerized feeder located in the free stall and forage was supplied via RIC bins (Insentec, Marknesse, The Netherlands). When lactating, cows received 1 kg/day of the standard lactation concentrate in the milking parlor. Forage composition did not differ among diets and was supplied ad libitum. For dry cows, forage consisted of grass silage, corn silage, wheat straw, and a protein source (rapeseed meal or soybean meal) in a ratio of 39:25:25:11 (DM basis). Post-calving, forage consisted of grass silage, corn silage, straw, and a protein source (rapeseed meal or soybean meal) in a ratio of 51:34:2:13 (DM basis). The ingredients, calculated chemical composition of concentrates, and the chemical composition of the diets based on the realized total feed intake were described earlier [10]. Diets were isocaloric [22] and equal in intestinal digestible protein and degraded protein balance [23].

### 2.3. Measure of Cow Routine Indicators

Cows’ body weight, milk yield, and dry matter intake data were measured from week 1 to week 4.

### 2.4. Collection of Blood Samples

Blood was sampled from the coccygeal vein into evacuated tubes (Vacuette, Greiner BioOne, Kremsmunster, Austria) containing EDTA at week 2 and week 4 post-calving per cow at 3 h before the morning feeding, referring to a previous study [24]. Samples were kept cold on ice for a maximum of 2 h until they were centrifuged at 2900× *g* for 10 min at 4 °C. Plasma was decanted, aliquoted, and frozen at −20 °C until the analysis.

### 2.5. LC–MS Measurements

Briefly, 100 μL of plasma was mixed with 100 μL of a phosphate buffer (pH = 7.0) and these samples were subsequently filtered to remove protein using a Pall 0.5 mL 10 kDa cut-off spin filter (Millipore Corp., Billerica, MA, USA) with centrifugation at 12,000 rpm for 15 min.

For the quantification of 39 metabolites, a targeted, standardized, and quality-controlled metabolomic profiling was performed using an LC-QQQ-MS analysis. Measurements were performed with a triple quadrupole mass spectrometer (Shimadzu LC-QQQ-MS; LCMS-8045) using the PFPP method as described earlier [25,26]. The sample injection volume was 1 μL, with an acquisition time of 28 min per sample. For more details, we refer the reader to Xu et al. [11,27].

### 2.6. Data Processing

#### 2.6.1. Metabolite Abundance Data

Metabolite abundances: built by using two-point corrected peak areas of metabolites detected by using LC-QQQ-MS after background removal. The 0 value of the metabolites was replaced with 1/100 of the metabolite’s minimum value. In total, 39 metabolites were detected including 21 amino acids and derivatives (alanine, arginine, Asparagine, aspartic acid, Citrulline, Cystine, glutamic acid, Glutamine, Glycine, Histidine, Isoleucine, Leucine, Lysine, methionine, phenylalanine, proline, serine, threonine, tryptophan, tyrosine, valine, Creatine, creatinine, 4-hydroxyproline, kynurenine, 2-Aminobutyric acid, carnosine, Cystathionine, and Methionine sulfoxide), 2 vitamins and derivatives (Pantothenic acid and Niacinamide), 2 fatty acids and derivatives (Acetylcarnitine and carnitine), 1 Alkaloid (cytidine), 1 Nucleoside (Thymidine), and 3 Other Organic Compounds (choline, Cyclic AMP, Allantoin).

#### 2.6.2. Ratios of Metabolite Abundances

Pairwise ratios of metabolite abundances (i.e., corrected peak areas) were calculated as follows:

$$r_{ij}=\frac{m_{i}}{m_{j}}$$

where *r_ij_* is the ratio of the abundance, *m_i_* and *m_j_* of the *i*-th and *j*-th metabolites, respectively. Before calculation, the 0 value of the metabolites was replaced with 1/100 of the metabolite’s minimum value taken across all samples. In total, 741 pairwise ratios were obtained from the 39 measured metabolites (see Section 2.6.1).

### 2.7. Statistical Analysis

#### 2.7.1. Exploratory Analysis

A principal component analysis (PCA) [28] was used to investigate patterns of plasma metabolites and ratios according to dietary treatment (lipogenic and glucogenic) and lactation week (week 2 and 4). Data were scaled to unit variance before the analysis.

#### 2.7.2. Discrimination and Classification Analysis of Plasma Metabolite Profiles

The XGboost algorithm [29] was used to discriminate and classify the cows’ metabolite profiles (using abundances and abundance ratios) according to diet (lipogenic diet or glucogenic diet) and lactation week (week 2 or week 4). Stratified sampling was used to divide data into a training set (containing 80% of the data) and a test set (containing 20% of the data) for model building and internal validation. The XGboost model was trained to two-group discrimination with diet grouping (lipogenic and glucogenic) and lactation as response variables. The models were built with a max decision tree depth of 4 splits and a learning rate of 0.1. Since in all the discriminations the number of cows of each group was exactly the same, we did not add any additional weighing to the samples and kept the weight of all samples the same. The quality of the predictive models was evaluated using the area under the curve of the receiver operating characteristic (AUC) curve, accuracy, sensitivity, and specificity of the model both on training and testing datasets, with the addition of every decision tree in the model and this measure was used for model optimization. The optimal model complexity, i.e., the number of trees used to build the model, was optimized by fitting predictive models with different numbers of trees to the training data and using the test data to assess the model quality. The algorithm stopped adding decision trees to the ensemble as soon as the model performance on the test dataset decreased irrespective of the increase in model performance on the training data. Once there were 50 decision tree additions with no increase in the AUC on test data, the algorithm was made to stop. The final model was the model with all the decision trees before the AUC on test data decreased.

The entire process of data splitting into training and test sets, model calibration, and validation was repeated 1000 times in order to account for sampling bias; the mean and 95% confidence interval (CI) values of the model performance variables (AUC, accuracy, sensitivity, and specificity) are reported [30].

#### 2.7.3. Variable Importance for Classification

Variable importance to the discriminant models built using the XGboost was quantified using the gain index, as a native quality measure for Gradient boosting regression and classification, which is calculated considering how many times a variable is selected for splitting, weighted by the squared improvement to the model as a result of each split, and averaged over all trees [31]. Basically, the gain quantifies the relative importance of each variable for the classification of a sample to one of the classes considered: lipogenic versus glucogenic and lactation week 2 versus lactation week 4.

**Figure 2:**
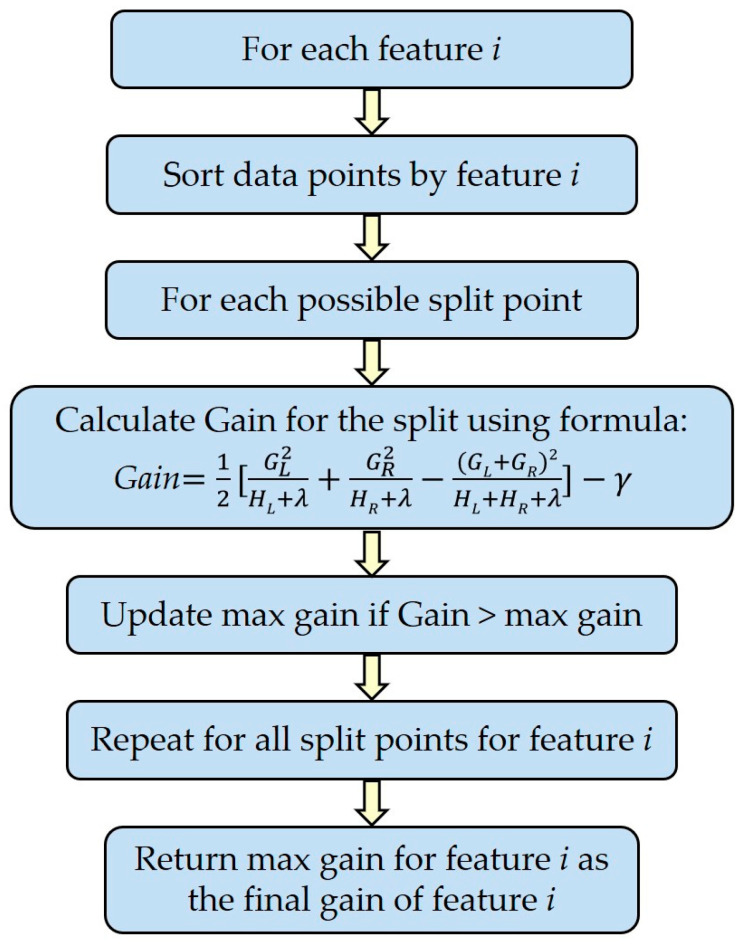
Overview of the calculation of the feature gain in the XGBoost algorithm. *G* is the sum of residuals; *H* is the number of residuals; subscripts *L* and *R* represent the left and right leaf, respectively; *λ* is the regularization parameter while calculating the similarity in the leaf to reduce overfitting. *γ* is the regularization on the next leaf. The pruning is performed based on whether or not gain is smaller than *γ*. (For readers who are interested in this, please visit https://xgboost.readthedocs.io/en/stable/index.html accessed on 1 April 2024).

We generated an importance matrix from the XGboost model that quantifies the contribution of each feature towards discrimination into diet or week and facilitates their prioritization based on their relative measure of gain (Figure 2). The gain implies the relative contribution of the corresponding feature to the model, calculated by taking each feature’s contribution for each tree in the model [32].

#### 2.7.4. Testing Metabolite Abundance and Metabolite Ratios between Dietary Intervention and Weeks

The top-10-ranking metabolite abundances and metabolite ratios (based on gain values from the XGboost algorithm) were statistically tested. Before testing, the data were tested for normality and homogeneity of variances. When the data are normally distributed and the variances are homogeneous, the Student’s *t*-test was used to test between groups; otherwise, the Wilcoxon test was used to test differences between groups.

#### 2.7.5. Software

For cows’ routine indicators (parities, body weight, milk yield, and dry matter intake), statistical analyses were performed using a repeated measures analysis in a mixed linear model [PROC MIXED, SAS version 9.3] (SAS Institute Inc., Cary, NC, USA, 2011) with the cow as the subject, considered as a random effect, for repeated weekly observations on individual cows. For metabolites and metabolite ratios, calculations and plots were performed in R (version 4.2.1). The package “factoextra” [28] was used for PCA. The package “xgboost” [29] was used for the XGboost model. The normal distribution test, homogeneity of variance test, Student’s *t*-test, and Wilcoxon rank sum test were all performed by R. The package “ggplot2” [33] was used for visualization.

## 3. Results and Discussion

### 3.1. Information of Cow Routine Indicators

Parity of the cows in the current study was 2.9 ± 0.2 vs. 3.1 ± 0.2 (*p* = 0.67) for the lipogenic and glucogenic diet, respectively. During the first 4 weeks of the lactation, cows had a milk yield of 40.3 ± 1.0 kg/day and 40.7 ± 1.2 kg/day (*p* ≤ 0.05), body weight of 662 ± 7 kg and 678 ± 7 kg (*p* = 0.76), and dry matter intake of 19.2 ± 0.4 kg/day and 20.4 ± 0.5 kg/day (*p* ≤ 0.05), for the lipogenic and glucogenic diet, respectively.

### 3.2. Overview of Metabolome Profiles for Cow Plasma

**Figure 3:**
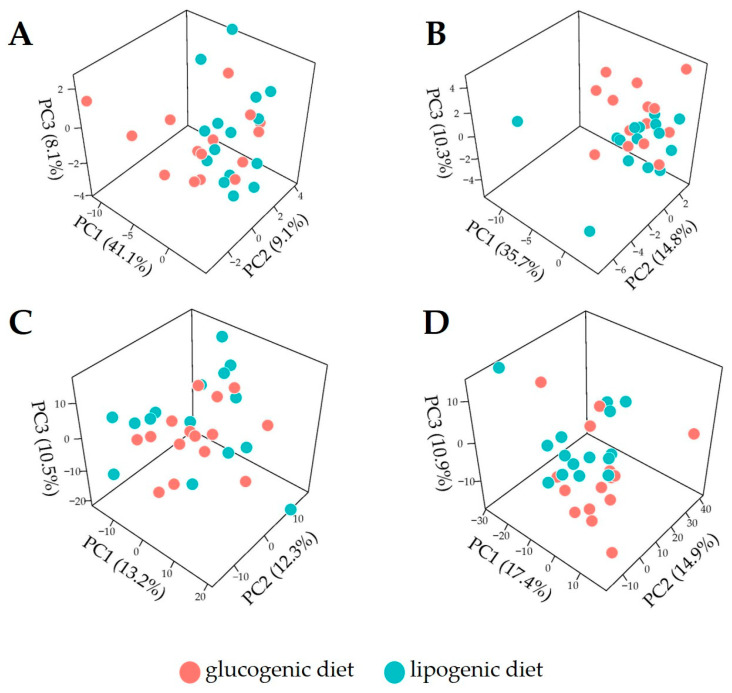
A principal component analysis score plot (3-dimensional) of plasma lipid profiles acquired in dairy cows fed with a glucogenic diet and lipogenic diet. (**A**) Week 2 metabolite abundances; (**B**) week 4 metabolite abundance; (**C**) week 2 metabolite abundance ratios; (**D**) week 4 metabolite abundance ratios; PC (principal component).

To explore the plasma metabolome data of cows fed the lipogenic and glucogenic diets and their effects, PCA was applied to metabolite abundance data or metabolite ratio data for week 2 and 4 separately. We observed weak dietary effects in lactation week 2 either using metabolite abundances (Figure 3A) or ratios (Figure 3C). PCA applied on data from week 4 (Figure 3D) showed a weak effect of diet discrimination when using metabolite abundance (Figure 3B) in lactation week 4, but a slighter better separation when metabolite ratios are used (Figure 3D). Overall, the exploratory analysis based on PCA suggests that changes induced by dietary intervention on plasma metabolites may be limited, so an unsupervised dimension reduction analysis is not sufficient to distinguish them. For this reason, we also applied a discrimination analysis by using the XGboost algorithm to build predictive models to discriminate between dietary treatments.

### 3.3. Discrimination of Dietary Intervention

**Figure 4:**
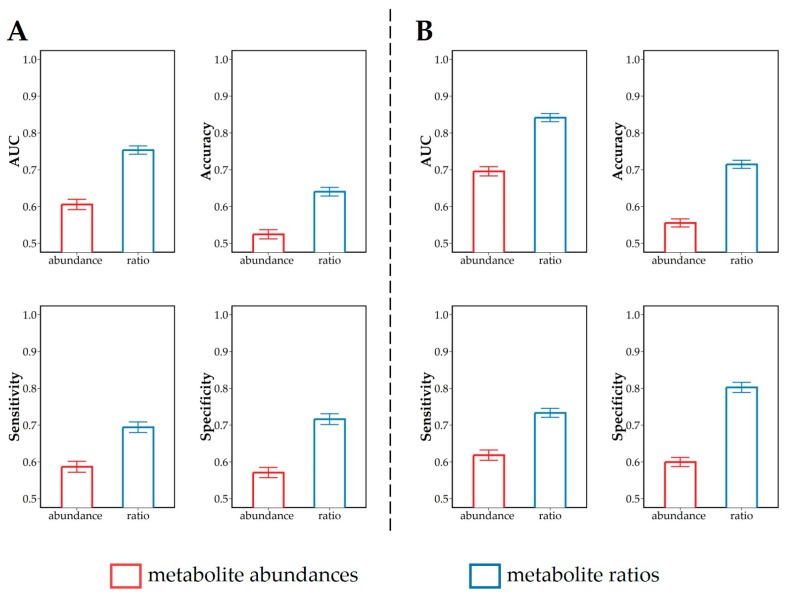
Quality measures (area under curve (AUC), accuracy, sensitivity, and specificity) of the predictive XGboost models used to discriminate the plasma metabolite abundances and ratios collected in cows fed with a lipogenic diet and glucogenic diet. Predictive modeling was performed per lactation week, separately; (**A**) week 2 and (**B**) week 4. Results are given as averages with the associated 95% confidence intervals calculated over the 1000 predictive models built.

Results of predictive modeling using the XGboost algorithm of the effects of lipogenic and glucogenic dietary intervention on plasma profiles are given in Figure 4, as average AUC, accuracy, sensitivity, and specificity with associated 95% confidence intervals. Discriminative models built using ratios (Figure 4A,B) were stronger than those built using the metabolite abundances (Figure 4A,B). When using metabolite ratios instead of abundances to discriminate diet in week 2, the average AUC increased from 0.61 to 0.75, the average accuracy increased from 0.52 to 0.64, the average sensitivity increased from 0.59 to 0.69, and the average specificity increased from 0.57 to 0.72, respectively. Similarly, using metabolite ratios instead of metabolite abundances to discriminate diet in week 4, the average AUC increased from 0.70 to 0.84, the average accuracy increased from 0.56 to 0.71, the average sensitivity increased from 0.62 to 0.73, and the average specificity increased from 0.60 to 0.80. These results indicate that the metabolite ratios possess a higher discrimination power compared with the metabolite abundances. This may be because the ratio between metabolites can reflect the relationship between metabolites compared with a single metabolite. Such changes in metabolite ratios in the present study point to perturbations between energy metabolic pathways such as lipid metabolism and amino acid metabolism. Table A1 summarizes the list of annotated metabolite pathway discrimination according to the KEGG pathway database (https://www.genome.jp/kegg/ accessed on 27 August 2022).

In comparison with the classification of metabolite profiles in week 2, classification in week 4 resulted in higher AUC, accuracy, sensitivity, and specificity values, indicating that the discrimination effect of dietary intervention on plasma metabolite profiles in week 4 (Figure 4B) is stronger than in week 2 (Figure 4A). We speculate that differences in the effects of diet on cows during early lactation increase gradually over time. During early lactation, increased feed intake cannot meet the cows’ rapidly increasing energy requirements for milk production, which result in a negative energy balance (NEB) [6,34]. According to van Knegsel et al. [10], the NEB value of lactating cows was lowest in week 2 after calving, and then gradually increased. The glucogenic diet alleviated NEB in early-lactation cows in a more pronounced manner than the lipogenic diet [10]. This difference in the effect of the diet may be responsible for the discriminative performance of week 4 being better than that of week 2.

### 3.4. Analysis of Relevant Metabolite Features Discriminating between Glucogenic and Lipogenic Diets

**Figure 5:**
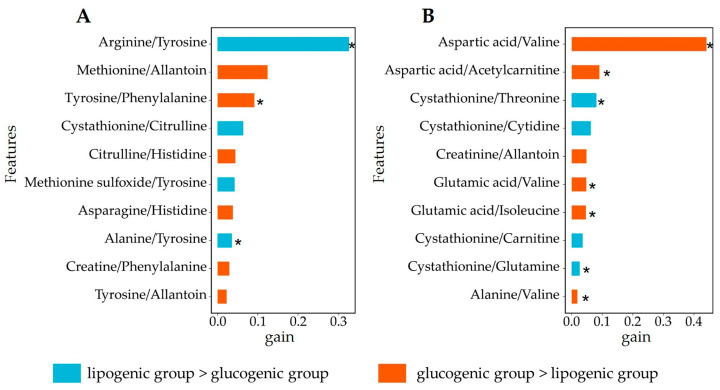
The top-10-ranking metabolite abundance ratios (based on gain values from the XGboost algorithm) that contributed the most to the predictive models used to discriminate between plasma profiles of cows fed lipogenic and glucogenic diets for (**A**) gain plots for the predictive modeling for samples collected in lactation week 2 and (**B**) gain plots for the predictive modeling for samples collected in lactation week 4. The gain quantifies the importance of a given metabolite–metabolite ratio to the prediction sample belonging to the two classes. * indicates that a particular metabolite abundance or ratio is different between diet groups (*t*-test, *p* ≤ 0.05).

Given its better discriminative performance (Figure 4), we considered for the further analysis only the model built using metabolite abundance ratios. We extracted the most relevant features to pinpoint the metabolite ratios, which contributed the most to the discrimination between lipogenic and glucogenic diets. Gain values for the top-10-ranking metabolite ratios are shown in Figure 5. Altered ratios between metabolites may indicate perturbations in specific metabolic pathways [18], and shed light on the metabolic mechanism underlying different dietary treatments.

Changes in amino acid concentrations are sensed by peripheral organs such as adipose tissue and the gut, and metabolic energy processes are controlled through these signals [35]. In week 2 of lactation, arginine/tyrosine (gain = 32.7%) was the top feature that contributed to the discrimination of lipogenic and glucogenic diets (Figure 5). Arginine/tyrosine was higher in the lipogenic group than the glucogenic group (*p* ≤ 0.05). According to a previous study, plasma arginine was positively correlated with the energy balance of dairy cows [36]. A glucogenic diet can lead to increased concentrations of plasma glucose and plasma insulin [24], and arginine may mediate this relationship. Arginine enhances glucose-induced insulin secretion, which is mediated by membrane depolarization [37]. Tyrosine synthesis is largely dependent on its conversion from phenylalanine, a nutrient for dairy cows derived from diet or ruminal microbes [38,39], in the liver of lactating bovines and glands of mammals [40,41]. Tyrosine is a precursor for the synthesis of adrenaline, which stimulates gluconeogenesis in the liver, helping in part to compensate for low glucose levels [42]. However, weak positive correlation was found between plasma tyrosine concentration and energy balance [36].

In week 4, aspartic acid/valine (gain = 44.1%) was the top feature that contributed to the discrimination of lipogenic and glucogenic diets. Aspartic acid/valine was significantly higher in the glucogenic group than in the lipogenic group (*p* ≤ 0.05). Aspartic acid is a glucogenic amino acid that can be used for hepatic gluconeogenesis in dairy cows [43], which usually occurs during the NEB stage [44]. Valine, as a branched-chain amino acid, can also be used for hepatic gluconeogenesis [45]. However, no significant correlation between plasma valine and EB was observed [36].

**Figure 6:**
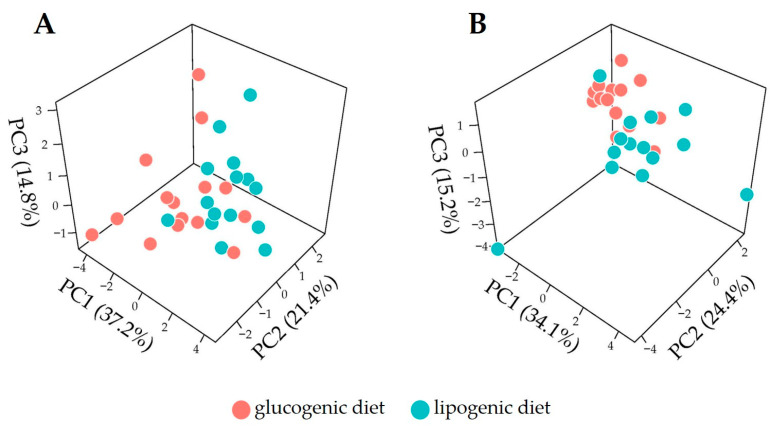
A principal component analysis score plot (3-dimensional) of plasma lipid profiles acquired in dairy cows fed with a glucogenic diet and lipogenic diet. (**A**) Week 2 metabolite abundance ratios; (**B**) week 4 metabolite abundance ratios. The top-10-ranked features were extracted from the XGboost discriminant-based feature importance matrix, which quantifies the contribution gain value of each feature to the discriminant as shown in Figure 5.

We performed PCA to explore and visualize the plasma profiles using only the top 10 most discriminant features presented in Figure 5. The PCA score plots given in Figure 6 show a much better separation between lipogenic diet and glucogenic profiles if compared to the PCA results shown in Figure 3 where all metabolite ratios were used. This confirms the discrimination power of the predictive models and suggests that diet treatment effects may be confined to a limited number of metabolites and ratios thereof.

Carnitine and its ratio to other metabolites were not the most important features to distinguish between lipogenic and glucogenic diets. In a previous study, the glucogenic diet could alleviate NEB of cows compared with a lipogenic diet by reducing milk fat content [10]. Carnitine is an energy-metabolism-related metabolite that is involved in fatty acid metabolism [27,46]. Based on our results, other metabolite ratios such as arginine/tyrosine or aspartic acid/valine can also provide accurate dietary discrimination information. However, this does not mean that carnitine-involved fatty acid metabolism is not unaffected by differential diets of dairy cows.

### 3.5. Comparison of Plasma Metabolite Features with Respect to Lactation Week

To further explore the effects of the lipogenic and glucogenic dietary treatment on the metabolism of dairy cows in early lactation, we compared, for each diet separately, the plasma metabolite profiles of week 2 and week 4.

**Figure 7:**
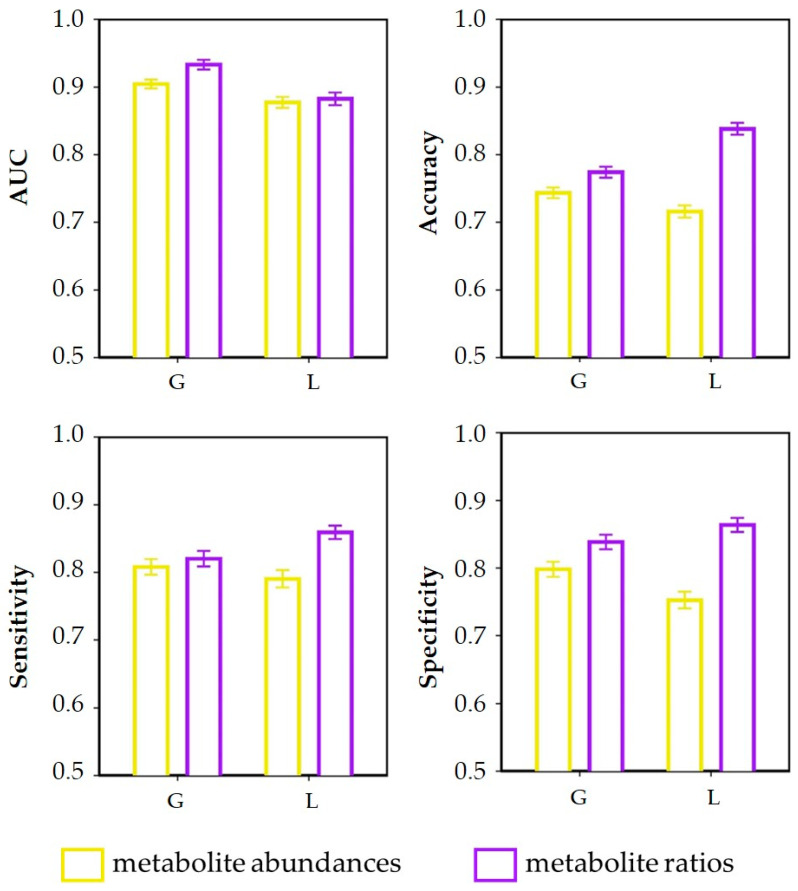
Quality measures (area under curve (AUC), accuracy, sensitivity, and specificity) of the predictive XGboost models used to discriminate the plasma metabolite abundances and ratios collected in cows during lactation week 2 and lactation week 4. Predictive modeling was performed per diet, separately; lipogenic diet (L) and glucogenic diet (G). Results are given as averages with the associated 95% confidence intervals calculated over the 1000 predictive models built.

**Figure 8:**
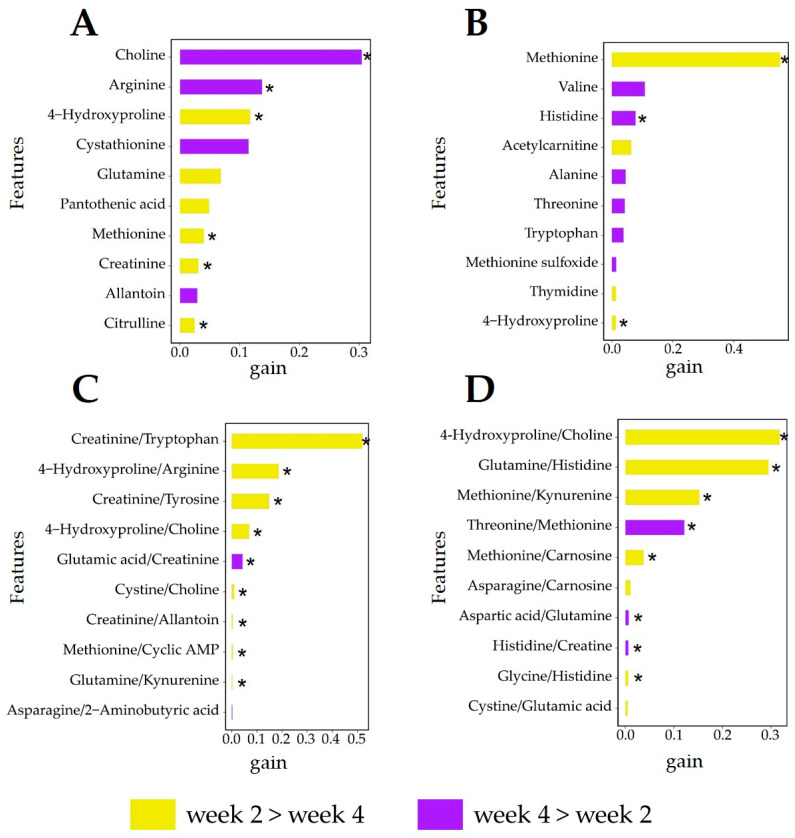
The top-10-ranking metabolite abundances and metabolite ratios (based on gain values from the XGboost algorithm) contributing the most to the predictive models used to discriminate between plasma profiles of cows in lactation week 2 and week 4 for the following: (**A**) Gain plots of metabolite abundance for the predictive modeling for samples collected from cows fed a lipogenic diet. (**B**) Gain plots of metabolite abundance for the predictive modeling for samples collected from cows fed a glucogenic diet. (**C**) Gain plots of metabolite ratios for the predictive modeling for samples collected from cows fed a lipogenic diet. (**D**) Gain plots of metabolite ratios for the predictive modeling for samples collected from cows fed a glucogenic diet. The gain quantifies the importance of a given metabolite or metabolite–metabolite ratio to the prediction sample belonging to the two classes. * indicates that a particular metabolite abundance or ratio is different between weeks (*t*-test, *p* ≤ 0.05).

The results for the predictive modeling given in Figure 7 show that a good discrimination can be obtained, for both diets, between the two lactation weeks with AUC values larger than 0.85: as observed in the case of the comparison of lipogenic and glucogenic diets (Figure 4), the use of metabolite ratios results in better predictive models than abundances. The top-10-ranked features are shown in Figure 8.

### 3.6. Analysis of Relevant Metabolite Features Discriminating between Lactation Weeks

For the lipogenic diet (Figure 8A,C), the top discriminating metabolite abundances (such as choline, arginine, and 4-hydroxyproline (hydroxyproline)) also appear as important discrimination ratios (such as 4-hydroxyproline/arginine and 4-hydroxyproline/choline). Although the feature contribution ranking of creatinine in the metabolite abundances was not as important as the above metabolites, its ratio to other metabolites was indeed an important discriminant feature, such as creatinine/tryptophan, creatinine/tyrosine, and glutamic acid/creatinine. The ratio of metabolites can provide multiple pieces of information of metabolites, which is related to the compound relationship between potential metabolic pathways, thereby discovering potential biomarkers [47].

Choline was the top feature for the discrimination of cows fed the lipogenic diet in week 2 and week 4 (Figure 8A). It was significantly higher in week 4 than in week 2 (*p* ≤ 0.05). Choline is a synthetic substrate for phosphatidylcholine, lysophosphatidylcholine, and sphingomyelin [48,49]. For one-carbon metabolism, choline and its oxidation product betaine can act as methyl group donors [50]. Due to the importance of choline itself in lipid metabolism, it will be offset when calculating ratios of choline with other metabolites. This is why the ratio of choline to other metabolites was not always the top one feature of the lactation week discrimination using the metabolite ratios. The ratio of creatinine to other metabolites such as tryptophan and tyrosine contributes significantly to lactation discrimination in lipogenic diets (Figure 8C). Energy balance is negatively correlated with plasma creatinine [36]. Plasma creatinine is associated with muscle protein mobilization when cows are in NEB [51].

For the glucogenic diet (Figure 8B,D), the top discriminating metabolite abundance for the prediction model of week 2 and week 4 was methionine, whose ratio to other metabolites like methionine/kynurenine, threonine/methionine, and methionine/carnosine also contributed to the week 2 versus week 4 discrimination. 4-Hydroxyproline/choline was the top feature for discrimination between lactation week 2 and week 4 of cows fed the glucogenic diet. It is significantly higher in week 2 than in week 4 (*p* ≤ 0.05).

Methionine is an essential substrate in one-carbon metabolism, and its intermediate metabolite, S-adenosylmethionine, is a universal methyl donor involved in many downstream cellular reactions [52]. The synthesis pathway of phosphatidylcholine involves two routes: (1) from choline (via the cytidine diphosphate–choline pathway) and (2) from phosphatidylethanolamine, in three consecutive methylation steps catalyzed by phosphatidylethanolamine methyltransferase with S-adenosylmethionine as the methyl donor [53]. Therefore, when the amount of choline available in the body is limited, the demand for methionine utilization will increase [54]. 4-Hydroxyproline in plasma has a negative relation with energy balance [36]. 4-Hydroxyproline/choline was higher in week 2 than in week 4 (*p* ≤ 0.05) and choline itself did not serve as an important feature in the discrimination of week 2 and week 4 (Figure 8B,D). This means that the NEB of week 4 is less obviously compared with that of week 2, which is related to the phosphatidylcholine synthesis pathway in which methionine participates.

## 4. Conclusions

In this study, we exploited plasma metabolite abundances and ratios in combination with machine learning to investigate the interaction between dietary management (glucogenic vs. lipogenic diet) and lactation week (week 2 vs. week 4 after calving). We found that the ratios of metabolite abundances better discriminate between dietary treatments in dairy cows in early lactation (week 2) compared with standard metabolite abundances. The metabolite ratios arginine/tyrosine and aspartic acid/valine may be potential biomarkers for distinguishing the metabolic effects of a lipogenic diet and glucogenic diet, which mainly involve the urea cycle and amino acid metabolism. Choline and the creatinine/tryptophan ratio may be potential biomarkers to discriminate a dietary effect in cows fed the lipogenic diet in week 2 or week 4. Methionine and the 4-hydroxyproline/choline ratio may be used to discriminate dietary effects in cows fed the glucogenic diet in week 2 or week 4. The energy metabolism of dairy cows with the lactation week is affected by a lipogenic diet and glucogenic diet, respectively, which may be related to different lipid metabolism pathways. This study supports the concept that metabolite ratios, not only abundance, can be used to explore the dynamics of molecular mechanisms and their relevance to the diet strategy of lactating dairy cows. Our study provides new insights into how to deeply analyze dairy metabolomic data.

## Data Availability

Dataset available on request from the authors due to ongoing research.

## Appendix A

**Table A1:** The list of annotated metabolite pathway classification.

Pathway	Total	Hits	Raw *p*	Impact
Arginine biosynthesis	14	5	1.49 × 10^−5^	0.42
Histidine metabolism	16	4	5.51 × 10^−4^	0.31
Alanine, aspartate, and glutamate metabolism	28	5	5.59 × 10^−4^	0.53
Glycine, serine, and threonine metabolism	34	5	1.41 × 10^−3^	0.30
Arginine and proline metabolism	38	5	2.36 × 10^−3^	0.29
Phenylalanine, tyrosine, and tryptophan biosynthesis	4	2	3.77 × 10^−3^	1.00
D-Glutamine and D-glutamate metabolism	5	2	6.17 × 10^−3^	1.00
Cysteine and methionine metabolism	33	4	9.03 × 10^−3^	0.32
Pantothenate and CoA biosynthesis	19	3	1.16 × 10^−2^	0.01
beta-Alanine metabolism	21	3	1.53 × 10^−2^	0.06
Phenylalanine metabolism	12	2	3.64 × 10^−2^	0.36
Glyoxylate and dicarboxylate metabolism	32	3	4.70 × 10^−2^	0.11
Pyrimidine metabolism	38	3	7.19 × 10^−2^	0.01
Glutathione metabolism	28	2	1.61 × 10^−1^	0.11
Tryptophan metabolism	41	2	2.86 × 10^−1^	0.24
Nicotinate and nicotinamide metabolism	13	1	2.89 × 10^−1^	0.19
Glycerophospholipid metabolism	36	1	6.14 × 10^−1^	0.03
Tyrosine metabolism	42	1	6.71 × 10^−1^	0.14
Primary bile acid biosynthesis	46	1	7.05 × 10^−1^	0.02

The “Total” is the total number of compounds in the pathway; the “Hits” is the actually matched number from the user uploaded data; the “Raw *p*” is the original *p*-value calculated from the enrichment analysis; the “Impact” is the pathway impact value calculated from the pathway topology analysis. Pathway annotation is in accordance with the KEGG pathway database (https://www.genome.jp/kegg/ accessed on 27 August 2022).
